# Inhibitory properties underlying non-monotonic input-output relationship in low-frequency spherical bushy neurons of the gerbil

**DOI:** 10.3389/fncir.2015.00014

**Published:** 2015-03-31

**Authors:** Thomas Kuenzel, Jana Nerlich, Hermann Wagner, Rudolf Rübsamen, Ivan Milenkovic

**Affiliations:** ^1^Department of Zoology/Animal Physiology, Institute of Biology II, RWTH Aachen UniversityAachen, Germany; ^2^Faculty of Biosciences, Pharmacy and Psychology, Institute of Biology, University of LeipzigLeipzig, Germany; ^3^Faculty of Medicine, Carl Ludwig Institute for Physiology, University of LeipzigLeipzig, Germany

**Keywords:** spherical bushy cells, gerbil, inhibition, rate-level function, temporal precision

## Abstract

Spherical bushy cells (SBCs) of the anteroventral cochlear nucleus (AVCN) receive input from large excitatory auditory nerve (AN) terminals, the endbulbs of Held, and mixed glycinergic/GABAergic inhibitory inputs. The latter have sufficient potency to block action potential firing *in vivo* and in slice recordings. However, it is not clear how well the data from slice recordings match the inhibition in the intact brain and how it contributes to complex phenomena such as non-monotonic rate-level functions (RLF). Therefore, we determined the input-output relationship of a model SBC with simulated endbulb inputs and a dynamic inhibitory conductance constrained by recordings in brain slice preparations of hearing gerbils. Event arrival times from *in vivo* single-unit recordings in gerbils, where 70% of SBC showed non-monotonic RLF, were used as input for the model. Model output RLFs systematically changed from monotonic to non-monotonic shape with increasing strength of tonic inhibition. A limited range of inhibitory synaptic properties consistent with the slice data generated a good match between the model and recorded RLF. Moreover, tonic inhibition elevated the action potentials (AP) threshold and improved the temporal precision of output functions in a SBC model with phase-dependent input conductance. We conclude that activity-dependent, summating inhibition contributes to high temporal precision of SBC spiking by filtering out weak and poorly timed EPSP. Moreover, inhibitory parameters determined in slice recordings provide a good estimate of inhibitory mechanisms apparently active *in vivo*.

## Introduction

Auditory brainstem circuits involved in sound source localization achieve extraordinary discharge accuracy through several mechanisms including the biophysical properties of the neurons (Trussell, 1999) and the integration of excitatory synaptic inputs with non-primary inhibition (Grothe, 2003). Spherical bushy cells (SBC) of the anteroventral cochlear nucleus (AVCN) receive strong excitatory input through giant synaptic terminals, the endbulbs of Held. Acoustically evoked inhibition critically contributes to signal processing in the AVCN (Shofner and Young, 1987; Ebert and Ostwald, 1995; Gai and Carney, 2008). It shapes the response tuning (Caspary et al., 1994; Kopp-Scheinpflug et al., 2002; Kuenzel et al., 2011) and helps to improve phase-locking precision (Dehmel et al., 2010) by raising the action potentials (AP) threshold (Kuenzel et al., 2011). Furthermore, the non-monotonic rate-level tuning reported for SBC (Winter and Palmer, 1990; Kuenzel et al., 2011) is likely caused by inhibition, as shown in gerbils by *in vivo* extracellular recordings in combination with pharmacology (Kopp-Scheinpflug et al., 2002).

SBC receive inhibitory inputs from multiple sources. Neurons in the ipsilateral deep dorsal cochlear nucleus (DCN) projecting via the tuberculoventral tract provide on-characteristic frequency inhibition (Wickesberg and Oertel, 1990; Campagnola and Manis, 2014). Moreover, neurons within the AVCN are a source of spatially broad inhibitory connections (Campagnola and Manis, 2014). Inhibitory postsynaptic currents (IPSCs) in SBCs of gerbils are rapidly rising and large, but exhibit slow decay time constants and activity-dependent reduction of the phasic amplitude and further prolongation of the IPSC decay (Nerlich et al., 2014a). While the glycinergic component dominates the inhibitory conductance, GABAergic transmission enhances the inhibitory strength and shapes its duration at physiologically relevant rates. Together, these properties cause an increased temporal summation of IPSC during ongoing activity. These data suggested that inhibition in SBC probably acts as a high-pass filter attuned by the overall stimulus level. In accordance with the data from SBC in gerbils, recent studies in mice suggested similar inhibitory properties (Xie and Manis, 2013, 2014).

However, the *in vivo* properties of inhibition and the time course of its action remained elusive, because inhibitory postsynaptic potentials were assessed neither by juxtacellular (Englitz et al., 2009; Kuenzel et al., 2011) nor by intracellular recordings (Paolini et al., 1997) of SBC. Apparently, the onset of inhibition is delayed with respect to the main excitatory input, as directly evidenced by electrical stimulation *in vivo* (Paolini and Clark, 1998) and indirectly by sound-stimulation (Kuenzel et al., 2011; Nerlich et al., 2014a).

Here, we seek to deduce the properties of acoustically evoked inhibition affecting the SBC in the intact brain. To this end, we modeled non-monotonic rate-level functions (RLF) in SBCs using spike arrival times recorded *in vivo* as model input. Our considerations are based on the assumption that the model parameters producing the closest match between data and model are a good estimate of the *in vivo* inhibitory properties. We constrained the inhibitory synapse model by the data recorded in brain slices. Furthermore, we explored the effects of inhibition on the temporal precision of SBC firing as observed *in vivo*.

## Materials and Methods

### *In Vivo* Recordings and Data Analysis

*In vivo* data used in this study were acquired from bushy cells located in the rostral AVCN of anesthetized gerbils, along with a larger dataset already published elsewhere (Kuenzel et al., 2011). Animals were anesthetized with an intraperitoneal injection of ketamine (80 μg/g body weight) and xylazine (Rompun, 12 μg/g body weight). All experimental procedures were in accordance with the European Communities Council Directive (86/609/EEC) and approved by a local animal ethics committee. The superior-anterior bulla chamber was opened and a craniotomy was performed through the superior semicircular canal. A custom probe containing an earphone speaker (SHURE SCL2) and an *in situ* microphone for calibration and compensation of the acoustic transfer function (G.R.A.S Type 40AG) was sealed onto the exposed ear canal. Animals were attached to a custom stereotactical device via a headmount glued to the skull. The rostral AVCN was accessed with low-impedance patch electrodes (5–7 MΩ; filled with, in mM: 126 K-gluconate, 20 KCl, 10 Na-Phosphocreatine, 4 Mg-ATP, 0.3 Na-GTP, 0.5 EGTA, 10 HEPES; pH adjusted to 7.2 with KOH; 310 mOsmol) using angles described by Frisina et al. (1982). Juxtacellular access to SBC was confirmed by spike signal amplitude (>2 mV) and at least fivefold increase in pipette resistance. Only units with discernable three-component complex waveform (Englitz et al., 2009; Typlt et al., 2010; Kuenzel et al., 2011) were included in the analysis. Sound stimuli (generated with MATLAB, presented through TDT System-II hardware PD1; PA4; HB5) were 50 ms cosine ramped pure tones of varying frequency and level, followed by 350 ms silence, ten repetitions each were presented. Sound-evoked responses of the juxtacellular potential waveform were recorded at 100 kHz sampling rate for offline analysis with a Multiclamp 700B patch amplifier (Axon) and custom software. Offline analysis yielded individual timing and metrics of the complex events: the presynaptic component (indicating the discharge of the endbulb terminal), the postsynaptic EPSP, and the postsynaptic (SBC) action potential. This approach allowed for direct comparison of the input (AN) and output (SBC spike) signals along with the extraction of AN spike arrival times. For a detailed description of the waveform analysis, see Kuenzel et al. (2011).

**Figure 1:**
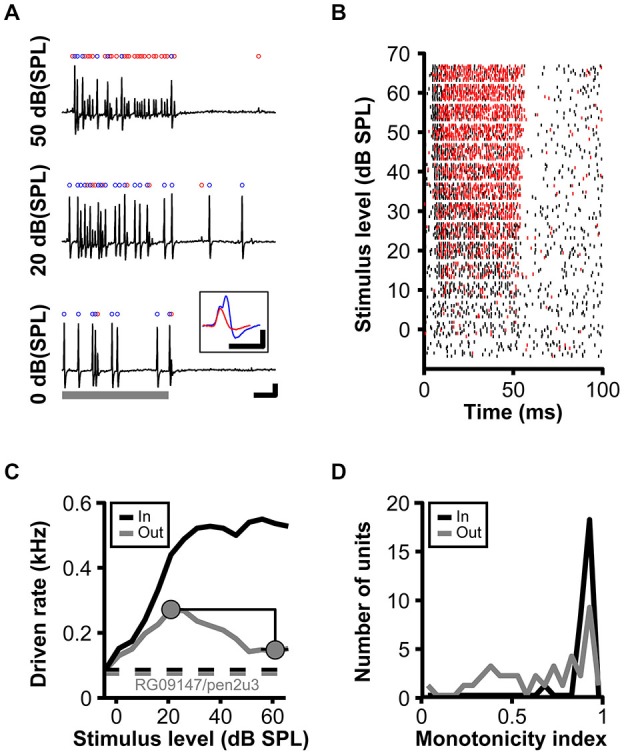
**Non-monotonic rate-level functions in gerbil low-frequency SBC. (A)** Juxtacellular recordings of a SBC (RG09147u3; CF = 861 Hz, MT = 0 dB, SR = 76 sp/s) upon pure tone stimulation at CF and increasing SPL. Stimulus duration indicated by the gray bar., scale bars: 10 ms/1 mV. The total number of input events (blue + red dots) increases with stimulus level, while the number of output spikes (blue dots) increases between 0–20 dB (SPL) and then decreases to 50 dB (SPL). Inset in **(A)** illustrates complex waveform shapes (scale bars: 1 ms/1 mV). **(B)** Rasterplot of spikes (black) and failures (red) during pure tone stimulation at CF and intensities −5–65 dB (SPL). Note that the number of spike failures increases in an intensity dependent manner during stimulus presentations (0–50 ms), but remains unchanged in the absence of acoustic stimulation (>50 ms). **(C)** Rate-level function for the same unit as in **(B)** (black line—input; gray line—output spikes). The respective dashed lines show spontaneous input and output rates. The monotonicity index is illustrated by gray circles. **(D)** Distribution of monotonicity indices for 37 low-frequency SBCs (black line input; gray line output spikes).

RLF were obtained for stimulation frequencies corresponding to the characteristic frequency of the respective units using at least 15 5 dB steps, starting 5 dB SPL below threshold. These data, comprising a subset of the data used for Kuenzel et al. (2011), were not systematically analyzed or presented before. In order to quantify the shape of the RLF, a monotonicity index (MI) was calculated by dividing the maximum response rate (peak rate) by the mean rate computed at the three highest presented sound levels (see Figure 1).

### Slice Preparation and Whole Cell Recordings

All experimental procedures were approved by the Saxonian district Government Leipzig (T 84/12, T 67/13) and conducted according to the European Communities Council Directive (86/609/EEC). Coronal slices (170 μm) containing the rostral AVCN were cut with a vibratome (Microm HM 650 V) from P22–P33 gerbils of either sex. At this stage, the development of inhibitory transmission onto SBCs was shown to be completed (Milenković et al., 2007; Nerlich et al., 2014a). Slicing was done in a low-calcium artificial cerebrospinal fluid (ACSF) solution containing (in mM): 125 NaCl, 2.5 KCl, 0.1 CaCl_2_, 3 MgCl_2_, 1.25 NaH_2_PO_4_, 25 NaHCO_3_, 25 glucose, 2 sodium pyruvate, 3 myo-inositol, 0.5 ascorbic acid, continuously bubbled with 5% CO_2_ and 95% O_2_, pH 7.4. Thereafter, the incubation of slices in the standard recording solution (ACSF as for slicing, except CaCl_2_ and MgCl_2_ were changed to 2 mM and 1 mM, respectively) was conducted for 30 min at 37°C. Recordings were made at nearly physiological temperature (33 ± 0.5°C).

Patch pipettes were pulled with Narishige PC-10 vertical puller from filamented borosilicate glass capillaries (Science Products) to have resistances of 4–5 MΩ when filled with (mM): 140 CsMeSO_3_, 20 TEA-Cl, 3.3 MgCl_2_, 10 HEPES, 0.1 EGTA, 5 QX-314-Cl, 5 phosphocreatine, 2 ATP disodium salt, 0.3 GTP disodium salt, and 0.2% biocytin or 50 μM ATTO 488 (pH 7.3 with CsOH). The recordings of synaptically evoked IPSCs (eIPSCs) on SBCs were conducted as reported earlier (Nerlich et al., 2014a). IPSCs were evoked from V_hold_ = −71 mV, by electrical stimulation of afferent fibers through a bipolar theta glass electrode (Sutter instruments, tip Ø 5 μm) filled with bath solution and placed at distances of 30–60 μm from the recorded cell. The stimulus intensity was slowly increased until obtaining stable IPSC amplitudes within a train. A stimulator (Master 8) was used to trigger single- or trains of pulse stimuli (100 μs), delivered via an isolated stimulus unit (AMPI Iso-flex). In all experiments, glutamate receptors were pharmacologically blocked (50 μM AP-5, 10 μM NBQX,). To rule out the possible effects of presynaptic GABA_B_ receptors located on inhibitory terminals (Lim et al., 2000), 3 μM CGP55845 was added to bath solution. Offline correction of voltages was done for 11 mV junction potential. The recordings were acquired with a Multiclamp 700B amplifier (Molecular Devices). The mean capacitance of the cells was 23.95 ± 5.54 pF (mean ± SD, *n* = 34). The average series resistance was 11.33 ± 1.66 MΩ (mean ± SD, *n* = 34), which was compensated by 50% to a remaining Rs of 3–7 MΩ. During experiments the series resistance changed on average by 1.5% (*n* = 34). Cells with series resistance changes >10% were excluded from analysis. There was neither a correlation between the IPCS amplitudes and decay time constants nor between the IPSC amplitudes and rise times (Nerlich et al., 2014a,b), thus ruling out the possible contribution of series resistance error to decay time constant measurements. Recorded signals were digitized at 50 kHz and filtered with a 6 kHz Bessel low-pass filter.

SBCs were verified according to their morphology revealed either on-line by intracellular labeling with ATTO 488 or by *post hoc* biocytin-labeling with Cy2-conjugated streptavidin (Milenkovic et al., 2009). Due to their large soma size and localization in the low-frequency area of the gerbil AVCN, these neurons can be visually distinguished from globular bushy cells. Image acquisition was done with a CCD camera (IMAGO Typ VGA; Till photonics) or with a confocal laser scanning microscope (TCS PS5, Leica), respectively. Data were examined with pClamp 10 software (Molecular Devices) and thereafter analyzed with custom-written Matlab routines. Mean peak amplitudes, 10–90% rise times and decay time constants were computed from averaged traces generated from >7 repetitions. IPSCs decay phase was fitted with bi-exponential function. The fitting was done in the range 95–5% of the peak IPSC amplitude. The weighted τ decay for bi-exponential fitting was calculated as τ_wd_ = (A_fast_ * τ_fast_ + A_slow_ * τ_slow_)/(A_fast_ + A_slow_), where A_fast_ and A_slow_ are amplitudes at *t* = 0 and τ_fast_ and τ_slow_ are the fast and slow time constants, respectively. IPSC conductance was calculated from the Cl^−^-driving force of 20 mV, determined by the V_hold_−E_IPSC_ = −71 – −50.8 mV (mean driving force = holding potential mean experimentally determined E_IPSC_). To exclude possible errors due to variations in series resistance, E_IPSC_ was controlled at the beginning of each experiment.

### Spherical Bushy Cell Model and Analysis

All simulations were performed with the NEURON simulation environment (Hines and Carnevale, 1997; Hines et al., 2009) with custom software written in Python 2.7, either under Linux ×86_64 or under Windows 7–64 bit. The biophysical properties of the SBC model were as previously published (Kuenzel et al., 2011). They were chosen to match the model by Rothman et al. (1993) with ion-channel models described in Rothman and Manis (2003). In the present study, the inactivating voltage-activated sodium conductance published in Rothman et al. (1993) was used, for which Marek Rudnicki (Technical University München) kindly provided the NMODL implementation.

A somatic compartment (*L* = 19.5 μm, *D* = 19.5 μm, Cm = 1 μF/cm^2^, g_Leak_ = 0.001 S/cm^2^), an axon hillock/first segment compartment (*L* = 15 μm, *D* = 4 μm, Cm = 1 μF/cm^2^, g_Leak_ = 0.001 S/cm^2^) containing all voltage-activated sodium conductance and a stretch of passive axon (*L* = 100 μm, *D* = 2 μm, Cm = 0.1 μF/cm^2^, g_Leak_ = 0.0001 S/cm^2^) were included in the model. Reversal potential for the leak conductance (g_Leak_ total: 14.5 nS) was −65 mV. Axial resistance was 150 Ω·cm for all compartments. Voltage-activated ion channel conductances were Na_v_ 1000 nS, LVA-K 200 nS, I_h_ 40 nS (E_rev_ −43 mV) and HVA-K 175 nS. Basic somatic parameters of the model SBC therefore resulted in a total membrane capacitance of 20.1 pF, a total input resistance (at rest) of 69.2 MΩ, and a resting membrane potential of −65.1 mV.

Simulations were run at a temporal resolution of ≤20 μs. The membrane potential of the model SBC was calculated and spikes were timed on the peak of the postsynaptic APs. The amplitude and relative timing of the EPSP and AP components were routinely analyzed using a waveform analysis procedure similar to the one described in Section *In Vivo* Recordings and Data Analysis. In the present simulation, waveform analysis was simplified by the fact that the arrival times of the presynaptic spikes are known. Postsynaptically evoked spikes were detected in a temporal window of −0.04 to +1.5 ms relative to the presynaptic spike by thresholding the first derivative of the membrane potential. As a reliable criterion for an AP, signal downward slopes ≥100 mV/ms (only occurring in the repolarization phase of the AP) were used. In the respective signals, the peak of the EPSP component was defined as the inflection point in the rising part of the waveform. This inflection point was calculated as the minimum between the local maxima in the first derivative (maximum EPSP and AP rising slope). The EPSP amplitude was defined as the difference (in mV) between the local baseline at the beginning of the analysis window and the peak of the EPSP component. In cases when APs failed, the EPSP amplitude was defined as the maximum membrane potential deflection in the analysis window and timing of the peak as well as upward and downward slopes were calculated.

### Synaptic Mechanisms

Two conductance point-processes representing on the one hand the excitatory endbulb of Held synapse (E_rev_ = 0 mV) and on the other hand the inhibitory inputs (E_rev_ = −75 mV) were connected to the somatic compartment of the SBC model. The E_rev_ for inhibitory current in our model matches the E_GABA_ experimentally determined with gramicidin perforated patch recordings (Milenković et al., 2007). Conductance traces were generated at the resolution of the simulation by convolving event templates with the input spike times. Input spike times were either taken from *in vivo* SBC recordings in gerbils (Section *In Vivo* Recordings and Data Analysis) or generated by a simple AN model (Section Auditory Nerve Model). The spike times driving the inhibitory point-process were delayed by 1 ms.

The conductance templates were fitted to match the rise time and decay time-constant obtained in patch-clamp recordings and scaled according to the event amplitude. The rise time (150 μs) and decay time-constant (*τ* = 200 μs) of the excitatory postsynaptic conductance (EPSG) template were fitted to EPSP waveforms recorded *in vivo* (Kuenzel et al., 2011).

In most simulations, no short-term depression of the endbulb inputs was implemented and EPSG amplitude was constant. In some simulations the EPSG template was randomly scaled for every event, resulting in a gaussian distribution of EPSG amplitudes ±0.2 standard deviations (“stochastic endbulb”). Finally, we also implemented a phenomenological depression model, as described before Nerlich et al. (2014a). Briefly, a depression state variable was reduced by every event and relaxed back to rest with a double exponential function (A_fast_ 0.75, A_slow_ 0.94, τ_fast_ 17.2 ms, τ_slow_ 57.0 ms). Note: the time-constants applicable for this state-model differ from recovery time-constants derived from paired-pulse experiments. The maximal EPSG amplitude was multiplied with the state variable at the time of the event resulting in the instantaneous maximal excitatory conductance. We tested a wider range of endbulb conductance for the dynamic endbulb models because both are expected to lower the excitatory efficacy. However, as shown by Pliss et al. (2009), endbulb conductances measured *in vitro* can span a large range of values, covering the range used in our model.

The template waveform of the inhibitory postsynaptic conductance (IPSG) was fitted to IPSC data from slice recordings presented in this study: The rise time was 455 ± 170 μs (*n* = 42), while the decay time-constant was 23.9 ± 5.5 ms (*n* = 41). In a number of experiments, the decay time-constant was systematically varied between 1 and 24 ms.

For the present simulations, temperature difference between the different sources of templates, i.e., *in vivo* and *in vitro* data, were not taken into account.

For the dynamic inhibitory synapse model, rate-dependent plasticity of the inhibitory synaptic mechanism was implemented as follows: Time- and input-dependent state-variables were used to simulate the dynamics of the model synapse. Input events caused instantaneous changes of specific magnitudes in the state variables, which relaxed back to their resting state following double-exponential relaxation functions (cf. Varela et al., 1997). Exponential parameters of the synaptic plasticity model were: A_fast_ 0.79, A_slow_ 0.99, τ_fast_ 31.1 ms, τ_slow_ 316 ms. Here, the exponential parameters A_fast_ and A_slow_ indicate the fraction of the synaptic conductance remaining after a given event. Note: IPSG amplitudes were not allowed to depress to zero, a minimal IPSG amplitude of 10% was enforced. Parameters of the decay tau change were: A_fast_ 8.7, A_slow_ 2.8, τ_fast_ 16.9 ms, τ_slow_ 151 ms, τ_max_ 80 ms. Here, the exponential parameters A_fast_ and A_slow_ represent the increase of the IPSG decay time constant per event in ms. All exponential parameters were extracted from recorded data by fitting model results to recorded data. From the state variables, we generated the amplitude and the decay time-constant for every event. The specific IPSG template waveform per event was generated prior to convolution.

### Auditory Nerve Model

A simple auditory nerve (AN) model was created to generate synthetic input spike trains for the SBC model. The AN model was implemented as a gamma-tone filterbank driving leaky integrate-and-fire neurons with noise and refractoriness (1.2 ms; threshold = 0.65) using the tools provided by the spiking neural-network simulator “Brian” (Goodman and Brette, 2008) under Python. Simulated sound stimuli were 2 ms ramped pure tones. Stimulus levels and output spike rates were adjusted to achieve output rates similar to the sustained part of AVCN responses. For the analysis of EPSP threshold (Figure 7), 5 s of sound stimulation was simulated for every condition. Here, both the CF of the simulated input fiber and the stimulus frequency were 5.5 kHz, to avoid phase-locking effects on ISI. For the analysis of phase-locking precision (Figure 8), 60 s of sound stimulation was calculated per condition. The CF of the simulated input fiber was 1.2 kHz.

**Figure 2:**
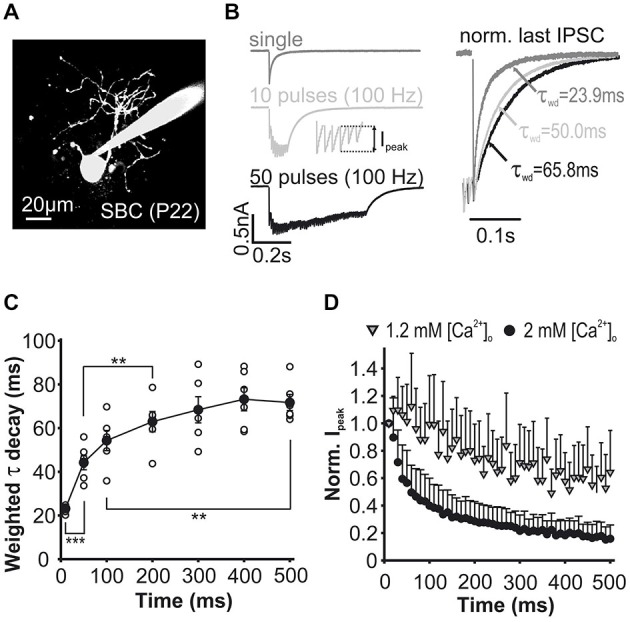
**Properties of inhibitory synaptic inputs to SBCs measured in slice recordings *in vitro*. (A)** Characteristic morphology of an SBC labeled with ATTO 488. **(B)** Left: Example traces of eIPSCs evoked by single or repetitive stimulation of synaptic inputs. I_peak_, foot to peak current amplitude as shown in inset. Right: Peak normalized eIPSCs showing prolongation of tau weighted (τ_wd_) during repetitive stimulation. **(C)** Weighted tau decay of the last IPSC in a train was prolonged during ongoing activity. Decay time constant was calculated for the single event and the last event in each train (10, 50; 100 Hz). Symbols indicate cells, **p < 0.01, ***p < 0.001. **(D)** Mean I_peak_ during trains of 50 pulses at 100 Hz normalized to the first IPSC under 1.2 mM and 2 mM extracellular calcium showing stronger depression under the latter condition.

**Figure 3:**
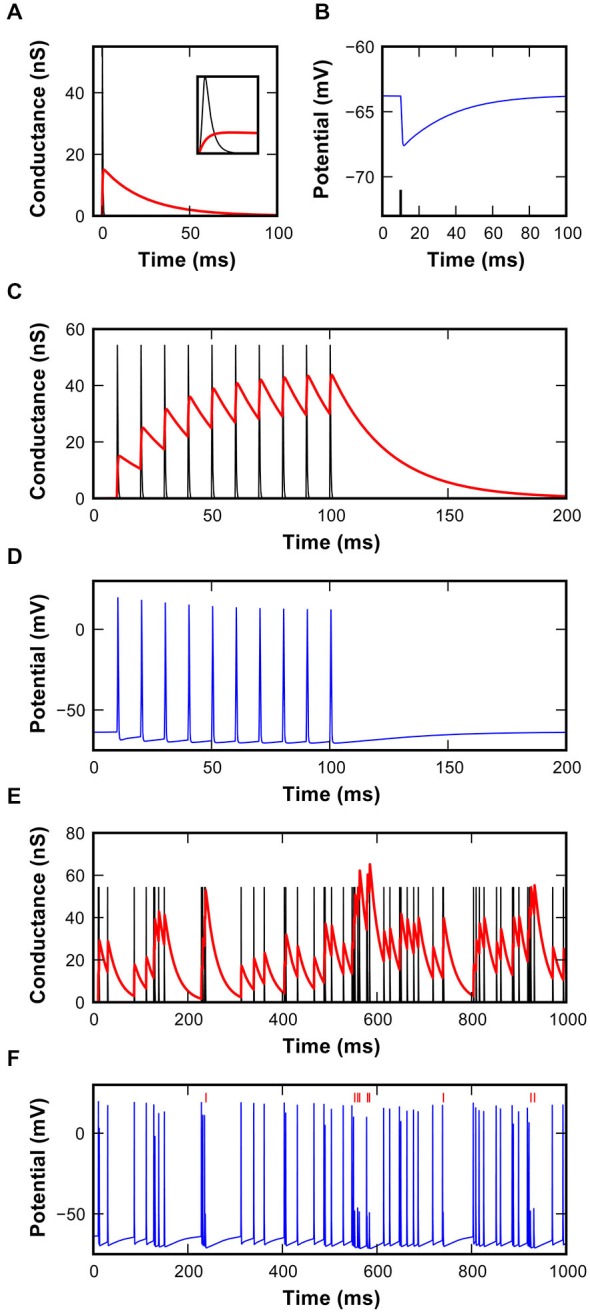
**Characteristics of the inhibitory synapse model. (A)** EPSG (black) and IPSG (red) waveforms used in the model. Inset shows magnification of the first 2 ms to reveal the rapid excitatory conductance. **(B)** Hyperpolarizing IPSP in the model resulting from the inhibitory conductance shown in **(A)**, triggered by the spike shown as a black line. **(C)** EPSG and IPSG resulting from 10 repetitions at 100 Hz using a static inhibitory model. Note the buildup of inhibitory conductance. **(D)** Simulated membrane potential resulting from the interaction of synaptic conductances shown in **(C). (E)** EPSG and IPSG during random activations of synaptic mechanisms at the average rate of 62 ± 7 s^−1^. Inter-event intervals follow a shifted-exponential distribution. **(F)** Simulated membrane potential resulting from the interaction of synaptic conductances shown in **(E)**. Failures identified by vertical red markers.

**Figure 4:**
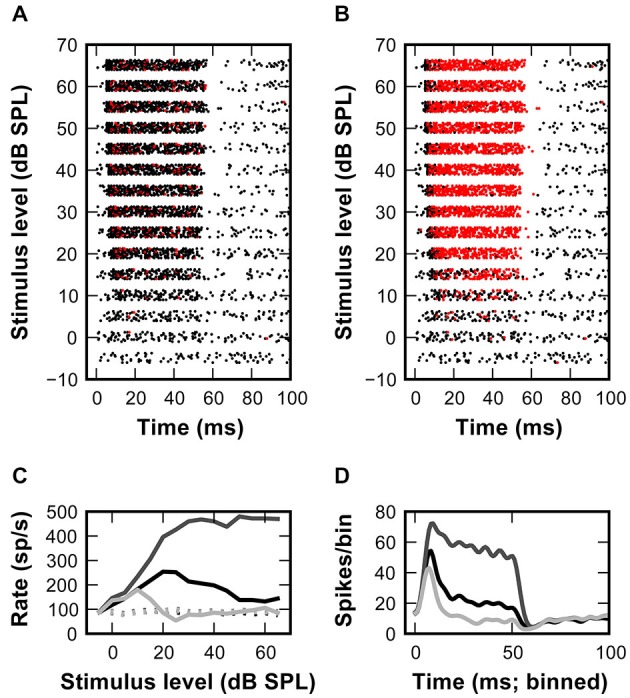
**Inhibitory inputs can account for non-monotonic rate-level functions in the SBC model. (A)** Rasterplot of the model response to a set of excitatory synaptic events elicited by the input spike times shown in Figure 1B (APs -black, failures—red). The model SBC follows the *in vivo* recorded AN input with minimal failures at all stimulus levels. **(B)** Adding inhibition to the model causes a non-monotonic rate-level function for responses at >15 dB. **(C)** Output rate-level functions calculated from the responses shown in **(A)** (dark gray line) and **(B)** (light gray line). Black line shows *in vivo* recorded data **(D)** PSTHs calculated from the data shown in **(C)**.

**Figure 5:**
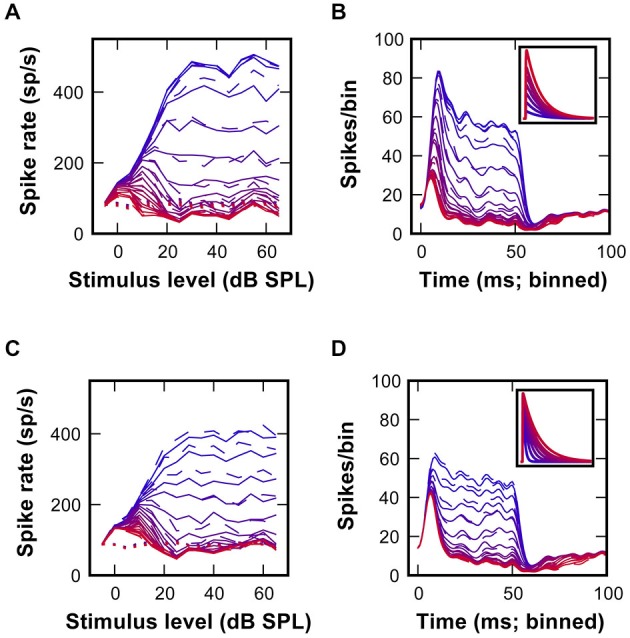
**Non-monotonicity of RLFs and phasic-tonic shape of PSTH depend on the strength of inhibition. (A)** Gradual changes of RLFs in a model SBC with increasing inhibitory conductance from 1 nS (blue) to 24 nS (red); decay time constant was fixed at 12 ms. Continuous lines are for matched spiketimes, dashed lines are for mixed spiketimes, dotted lines show spontaneous rate. **(B)** Gradual changes of model PSTHs resulting from the variations of inhibitory conductance shown in **(A)**. Inset shows the inhibitory EPSG waveform for simulations in **(A,B). (C)** Output rate-level functions obtained after employing an inhibitory conductance of constant amplitude (12 nS), but with tau decay between 1 ms (blue) and 24 ms (red). Line styles as in **(A). (D)** Average peri-stimulus time histograms for the data shown in **(C)**.

**Figure 6:**
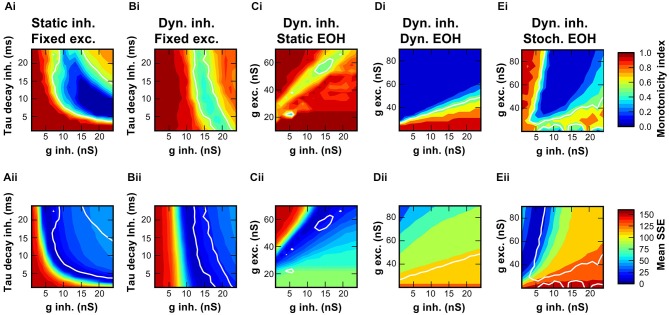
**Range of synaptic properties causing non-monotonic rate-level functions in the SBC model. (Ai,Bi)** Maps of monotonicity indices of RLF calculated from simulated responses for 256 combinations of inhibitory conductances (1–24 nS) and decay time constants (1–24 ms). Monotonicity indices are color coded ranging from dark red (MI = 1; monotonic) to dark blue (MI = 0; non-monotonic). White line show the conditions where the MI of the model equals the MI of the recorded unit. Simulations in A were performed with static inhibitory synaptic models, simulations in **(B)** with the dynamic inhibitory synapse model. **(Ci)** Amplitudes of excitatory and initial inhibitory conductances were varied in the dynamic synapse model, initial tau-decay was 24 ms. **(Di)** Simulation in **(Ci)** repeated with short-term depression of the endbulb included in the model. Note the wider range of excitatory conductance that was tested. **(Ei)** Simulation in **(Di)** repeated with stochastically varying endbulb conductance. **(Aii–Eii)** Maps of similarity between the model and the data for each condition in simulations are shown in **(Aii–Eii)**. Low SSE reveals a good match between the model and the data (dark blue), high SSE indicates a large difference (dark red). White contour line same as in **(Ai–Ei)**.

**Figure 7:**
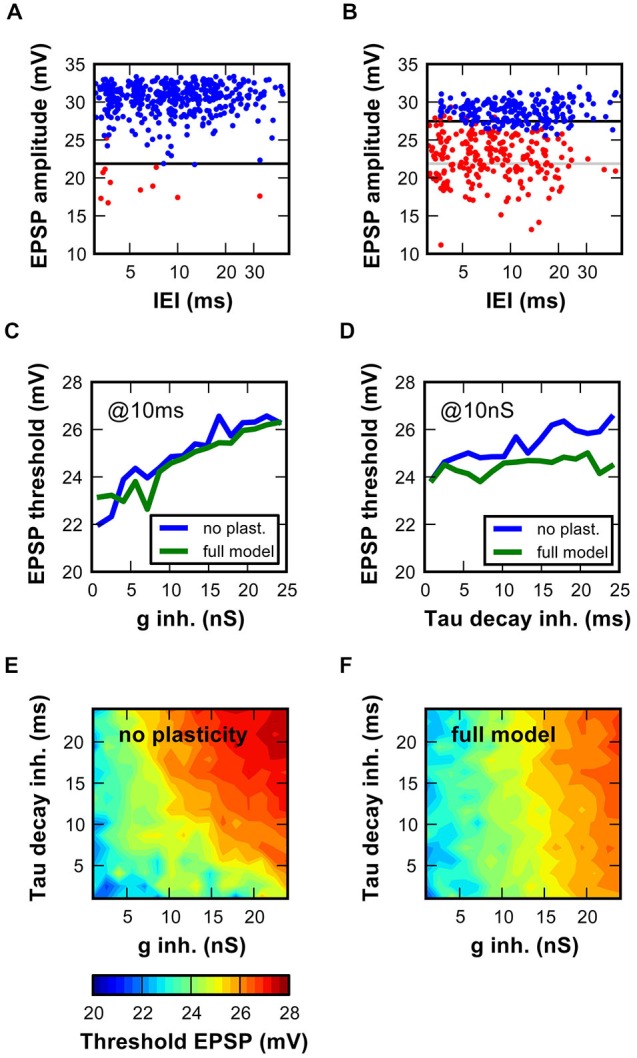
**Activity-dependent inhibition in the SBC model increased the EPSP amplitude necessary to initiate an AP. (A,B)** Measurement of threshold EPSP in the SBC model. EPSP component amplitudes of successful (blue) and failed (red) events are plotted against inter-event intervals. Optimal boundary between red and blue data points was used to estimate the threshold EPSP. **(A)** Simulations with low (1 nS) and **(B)** high (24 nS) inhibitory conductances. Note the threshold increase with stronger inhibitory conductance (black vs. light gray line). On average 525 ± 1.5 events were analyzed per condition. **(C,D)** EPSP threshold depends on inhibitory conductance characteristics (**C**, g_inh_ 1–24 nS, decay tau fixed at 10 ms; **(D)**, decay time constant 1–24 ms, initial inhibitory conductance of 10 nS). The blue and green lines respectively show data for the static and dynamic synapse models. **(E,F)** Threshold EPSPs calculated for 256 combinations of inhibitory conductance (1–24 nS) and inhibitory decay time-constant (1–24 ms). Threshold EPSP ranged from 20 mV (blue) to 28 mV (red). Simulations in **(E)** are performed with the static synapse model, simulations in **(F)** with the dynamic synapse model.

**Figure 8:**
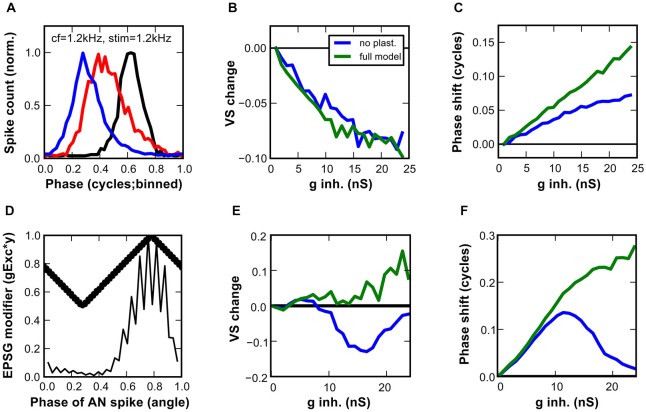
**Impact of inhibition on the temporal precision of output spikes in the SBC model. (A–C)** Responses of a model SBC to simulated sound stimulation at CF (1.2 kHz). **(A)** Cycle histograms of the input events (black), and the output events with low (blue; 1 nS) or with high (red; 24 nS) inhibitory conductances. **(B)** Differences in vector strengths between input and output spikes in the model with the static (blue) and dynamic (green) inhibitory conductances. A gradual increase in inhibitory conductance worsened the temporal precision. **(C)** The effect of the static (blue) and the dynamic (green) inhibition on the phase-shift, expressed as the difference between preferred phases of input and output spikes in the SBC model. **(D–F)** A proposed mechanism of an increase in temporal precision engages activity-dependent inhibition and phase-dependent excitation. **(D)** Phase-dependent EPSG modifier (thick line) superimposed on a cycle histogram of simulated AN inputs to the SBC model (thin line). The resulting EPSGs are up to 50% smaller at maximum deviation from the preferred phase phi. **(E)** The vector strength, and **(F)** the phase shift obtained in the SBC model that integrated phase-dependent EPSG with the static (blue) or dynamic (green) inhibition.

## Results

### Non-Monotonic Rate-Level Functions in Low Frequency SBC

The *in vivo* data analyzed and presented here were acquired along with a dataset published before (Kuenzel et al., 2011). The rationale of the present study was to investigate how acoustically evoked inhibition determines the firing properties of SBCs over a range of sound stimulus intensities, an aspect only briefly discussed earlier. Recordings from low-frequency SBCs commonly encountered non-primary like tuning with increased AP failure rates (defined as isolated eEPSP not followed by an eAP, Figure 1A, *red dots*) at higher sound pressure levels (Figure 1; Kuenzel et al., 2011). Still, the SBC onset response remained largely unaffected by the increasing SPL as can be seen from raster plots differentiating between eEPSPs and eAPs (Figure 1B). RLF for the SBC output at the units CF show a non-monotonic shape (i.e., an increase up to a maximum response rate at medium intensities followed by a decrease of response rate towards higher intensity levels; Figure 1C). It was hypothesized earlier that the non-monotonicity of the RLF is caused by the interaction of acoustically evoked inhibitory and excitatory inputs to SBCs (Kopp-Scheinpflug et al., 2002; Kuenzel et al., 2011).

To capture the shapes of the RLF, monotonicity indices (MI) were calculated by dividing the mean response rates at the respective three highest stimulus levels by the maximum response rates (Figure 1C). Thus, primary-like RLF tuning, typical for the AN input to the SBC, will be indicated by MI close to one, while lower values quantify the degree of non-monotonicity. For the example unit (RG09147pen3u3) presented in Figure 1, the MI were 0.98 (= 538/550) for the input and 0.54 (= 147.33/272) for the output. Across all units, the MI for the AN inputs was on average 0.95 ± 0.05 (*n* = 37), with only one unit ranging below 0.9, whereas the mean MI of the SBC output was 0.69 ± 0.26 (*n* = 37). From the latter, 30% (11/37) showed MI above 0.9 (i.e., monotonic RLF) and 46% (17/37) indices below 0.75 (i.e., strongly non-monotonic RLF; Figure 1D). To evaluate the potential contribution of inhibitory inputs to the range of shapes of SBC output RLFs, model calculations were employed using the timing of the synaptic events measured *in vivo* as inputs.

### Properties of Inhibitory Synaptic Events in Gerbil SBC

In order to ascertain a realistic model representation of the properties of inhibitory events, patch-clamp recordings were performed in acute brainstem slices containing the rostral AVCN of P22–33 gerbils (Figure 2). Synaptically evoked IPSCs in identified SBCs (Figure 2A) were triggered by electrical stimulation of afferent fibers. Single IPSCs (Figure 2B, *dark gray trace*) evoked in naïve synapses had an average conductance of 23.8 ± 5.4 nS (*n* = 41), a rapid 10–90% rise time (0.46 ± 0.18 ms) and a slow decay time constant (tau weighted) of 23.9 ± 5.5 ms (*n* = 41). Repetitive electrical stimulation of inhibitory inputs (10 pulses, 100 Hz) led to summation of the overall current (Figure 2B, *light gray trace*), while individual phasic events (inset) were subject to activity dependent changes in amplitudes. A prolongation of the stimulus train (50 pulses, 100 Hz) yielded a characteristic temporal profile of the total inhibitory current described by a transient temporal summation of individual events and progressive activity dependent amplitude decrease (Figure 2B, *black trace*). The activity dependent changes also included the prolongation of the decay time constant of the last IPSC (Figures 2B right,2C) from 23.1 ± 0.7 ms (single) to a maximum of 73.2 ± 5.3 ms reached after 40 pulses (Figure 2C, RM ANOVA, *n* = 6). In the course of a pulse train, phasic IPSC amplitudes (Ipeak) showed a depression that followed a double-exponential course when the recordings were performed using the standard extracellular solution containing 2 mM Ca^2+^ (Figure 2D, *black circles*; *n* = 24). During the first 5–6 events of a 100 Hz pulse-train, the phasic IPSC amplitude rapidly depressed below 50% of the initial value, with a more moderate reduction thereafter towards a steady-state phasic current of about 20% (18 ± 2%) of the initial values. Yet, the depression of phasic IPSC amplitudes was markedly different in extracellular calcium condition (1.2 mM), which is thought to more faithfully reflect the *in vivo* condition (Figure 2D, *gray triangles*; *n* = 6 cells) (Borst, 2010).

In 1.2 mM external Ca^2+^, the total inhibitory conductance was smaller at the stimulation onset, while towards the end of the train the baseline IPSCs were not different for the different calcium concentrations (conductance 1. IPSC: 1.2 mM = 17.9 ± 4.6 nS; 2 mM = 30.9 ± 5.9 nS, *p* < 0.001; max conductance within train: 1.2 mM 10–12th IPSC = 44.0 ± 8.2 nS; 2 mM 7–9th IPSC = 61.30 ± 6.1 nS, *p* < 0.01; conductance 48-50th IPSC: 1.2 mM = 30.1 ± 6.5 nS; 2 mM = 29.7 ± 6.0 nS, *p* = 0.88; *n* = 6, RM ANOVA, data shown in Nerlich et al., 2014b). Under lower calcium concentrations, phasic current amplitudes slightly increased to 110% during the first five stimulus pulses and thereafter gradually decreased towards a steady of about 60% (58 ± 24%) of the initial values (Figure 2D). Notably, single IPSCs and short trains (10 pulses at 100 Hz) showed faster decay time constants under the lower release probability condition, whereas longer and high frequency stimulation revealed similar tau decay in the two calcium conditions (1.2 vs. 2 mM Ca^2+^: single, 100 Hz 10th IPSC *p* < 0.05, 100 Hz 50th IPSC *p* = 0.72, 333 Hz 10th *p* = 0.27 and 50th IPSC *p* = 0.33, *n* = 11, RM ANOVA, data shown in Nerlich et al., 2014b).

Taken together, the activity dependent prolongation of the decay time constant and reduction in the IPSC phasic amplitudes endow the overall inhibitory current with complex temporal dynamics. As shown by our experiments in reduced extracellular calcium, the actual inhibitory strength in the intact brain may be lower than expected from the slice recordings. This warranted a systematic analysis of a range of different inhibitory conductances in the model. The possible contribution of such dynamic inhibitory profiles to the non-monotonic intensity tuning in SBCs was further explored by the implementation of the respective parameters into the SBC model.

### Non-Monotonic RLF Caused by Inhibition in the SBC Model

In the model, the IPSG waveforms were fitted to inhibitory events measured in the slice recordings detailed above, and the EPSG parameters were set to produce eEPSP waveforms recorded *in vivo* (Kuenzel et al., 2011; Figure 3A). The IPSG evoked a hyperpolarizing, long-lasting IPSP (Figure 3B). Due to the long decay-time constant, inhibitory conductance strongly summated in trains of events (Figure 3C), generating an increasing level of inhibition, as seen in whole-cell recordings. Surprisingly, due to the powerful endbulb EPSG (55 nS), summation of inhibition did not necessarily result in suppression of spiking in regular 100 Hz trains of excitation (Figure 3D). However, simulation of spontaneous activity with a shifted exponential distribution of intervals intermittently led to stronger summation of inhibition that indeed caused AP failures (Figures 3E,F). In the model, a maximum inhibitory conductance of 68.3 nS was allowed based on the respective peak values recorded at high stimulation frequencies in our slice experiments.

When spike arrival times of the AN input from *in vivo* recordings (Figure 1) were used to drive the excitatory synaptic mechanism with a fixed EPSG and no inhibition, only few AP failures occurred even at highest simulated sound pressure levels (Figure 4A, *red dots*). In fact, failures only occurred with short intervals, presumably when approaching the refractory period of the SBC. Under these conditions, the output of the SBC model without inhibition closely resembled the input (Figure 4C). Also, while the simulation shows the primary-like shape of the peristimulus time histogram (PSTH), the sharp distinction between the onset and ongoing response—phase typically seen in SBCs—is not sufficiently highlighted (Figure 4D).

Next, the same AN input events from *in vivo* recordings were used to additionally drive inhibitory synaptic mechanisms with a delay of 1 ms to the excitatory input. Under this condition numerous spike failures were observed especially during the sustained period of the stimulus response and predominantly at higher sound pressure levels (Figure 4B). This indicates less effective AP inhibition at low stimulus levels which also becomes evident in the non-monotonic profiles of simulated RLFs (Figure 4C) and in the prominent onset phase in the PSTH (Figure 4D). These simulation results are in agreement with an increase of inhibitory strength with increasing input rates at higher stimulus intensity levels.

Differences between the simulated (Figure 4C) and recorded RLF profiles (Figure 1C) and the respective PSTH-profiles (Figure 4D; Kuenzel et al., 2011) point to a suboptimal setting of inhibitory parameters in the model. Thus, we systematically varied the conductance of individual inhibitory events while keeping the decay time-constant fixed (Figures 5A,B). This conductance increase progressively transformed the RLF from a monotonic to a non-monotonic shape (Figure 5A). However, the respective change was paralleled by a progressive decrease of the onset component in the phasic-tonic PSTH (Figure 5B), which is inconsistent with *in vivo* recorded PSTHs. Next, the inhibitory strength was varied by systematically increasing the decay time-constant while keeping the conductance fixed (Figures 5C,D). This caused a comparable gradual transformation of RLF shapes (Figure 5C), but had a less prominent effect on the onset phase of the response (Figure 5D). To quantitatively compare the modeling results and the experimental data, we calculated the sum of the squared errors (SSE) between simulated and measured RLFs and PSTHs. With increasing inhibitory strength, either by varying inhibitory conductance or decay time-constant, the SSE rapidly declined to minima at conductances of 7.1 nS for RLF and 8.6 nS for PSTH (at 12 ms decay-tau) and a decay time-constant of 7 ms (at 12 nS conductance). These data suggest that strong and at the same time slow IPSG is a necessary condition for the occurrence of non-monotonic RLFs in SBC.

Initial simulations utilized a fixed temporal structure of inhibition delayed by 1 ms to the excitation. This was however only a first approximation of the actual *in vivo* condition. Next, the simulations were conducted with shuffled temporal relation between excitatory and inhibitory spike trains. This had only negligible effects on RLFs, PSTHs and SSE (dashed lines in Figures 5A–D). A comparison of the response rates from all repetitions for the matched and the shuffled condition showed a linear fit between the respective results (*y* = 0.99x + 1.7, *r*^2^ = 0.93; data not shown). Thus, we concluded that for the non-monotonic SBC output the overall level of summed inhibition was more important than the precise temporal structure of inhibitory events.

To assess the mutual contribution of IPSG and tau decay to RLF, the MI as a measure of the RLF shape and the SSE between the model and the data were calculated for many conductance and decay-tau combinations (Figures 6A,B). For the static synapse model without inhibitory plasticity, a broad range of combinations of inhibitory conductance and decay-tau values produced non-monotonic RLFs (Figure 6Ai). Plotting the MI assessed from *in vivo* data (MI = 0.54) as a contour in the plot revealed a wide range of parameter combinations that yield such results. However, the sum of squared errors between measured RLF and model RLF (Figure 6Aii) is minimal only for a subset of combinations. The best match with the recorded data was thus seen for an inhibitory conductance above 8 nS and a decay tau below 15 ms.

In slice recordings, the synaptically evoked inhibitory currents showed a prominent activity-dependent depression of the IPSC amplitudes and prolongation of tau-decay (Figure 2; also see: Nerlich et al., 2014a). Hence, the model was extended to include the two rate-dependent phenomena (Figure 6Bi). Despite the fact that a wide range of combinations of conductance- and decay-tau values produced non-monotonic RLF both in the static and the dynamic model, distinct differences are noteworthy: The MI obtained by the dynamic model were ≥0.3, whereas the static model also yielded values close to zero. Furthermore, in the dynamic model an initial inhibitory conductance of 13–17 nS produced a RLF matching the MI of the measured RLF with minimal SSE (Figure 6Bii) largely independent of the initial values of decay-tau. The respective inhibitory strength was within the range of conductances measured in our slice experiments under physiological extracellular calcium concentration (see Section Properties of Inhibitory Synaptic Events in Gerbil SBC).

Next, we addressed the question how the excitatory endbulb conductance influences the outcome of the RLF simulations, since it was shown earlier that the relative strength of excitation and inhibition controls the SBC output (Nerlich et al., 2014a). Here, we modeled the integration of excitation and inhibition particularly considering the non-monotonic RLF using a dynamic inhibitory input and a static endbulb (see Section Discussion). Under these conditions, only a confined range of combinations of excitatory and inhibitory conductances was able to produce non-monotonic RLFs (Figure 6Ci) with minimal difference between the measured data and the model (Figure 6Cii). The effective range of values that reproduced RLF most similar to the one measured *in vivo* was 52–62 nS for the excitatory conductance and 12–17 nS for the inhibitory conductance.

We next repeated the simulation with a dynamic model of the excitatory input that featured short term depression. A wide range of initial excitatory and inhibitory conductances yielded RLF of the same MI as the recorded data (Figure 6Di), however the model RLF were very different from the RLF obtained from the recorded data (Figure 6Dii). Thus, it seems unlikely that the inclusion of short-term depression in the model provides a valid description of the *in vivo* data.

Finally, we tested how the excitatory conductance influences the outcome of the simulation when stochastically varying endbulb conductance (mean EPSG ± 0.2 SD) was used. A wide range of MI was encountered (Figure 6Ei) including the MI of the recorded data. For mean endbulb conductances >55 nS the model RLF matched the recorded data with minimal error (Figure 6Eii). Under these conditions, the inhibitory efficacy seems to be larger, and an initial inhibitory conductance as low as ~7 nS provided the best match of the model to the recorded data.

These simulations show that, given a specific and confined range of excitatory and inhibitory synaptic properties are used, the SBC model with dynamic inhibition can closely reproduce RLF measured in the intact gerbil brain.

### Effect of Inhibition on the SBC Model Output: Threshold EPSP

In an earlier study (Kuenzel et al., 2011) it was shown with a simpler SBC model that hyperpolarizing inhibition may control the input-output function of SBC by increasing the threshold EPSP. The threshold EPSP was defined as the excitatory synaptic strength needed to elicit a spike. Here, we expand these findings by employing the complex model with physiological inhibitory parameters while using the “stochastic endbulb” (mean EPSG = 55 nS ± 9 nS; see Figure 6E). SBC event waveforms generated by the model were analyzed as described earlier (Kuenzel et al., 2011).

In a model with minimal inhibition (Figure 7A; 1 nS), AP failures occurred rarely and were caused by small EPSPs. The EPSP amplitude required to reach the AP threshold was 22 mV. The increase of the inhibitory conductance to 24 nS caused numerous failures (Figure 7B), due to the following reasons: (i) the overall EPSP amplitude was reduced especially at short inter event intervals, likely due to shunting. (ii) The threshold EPSP was increased by 5 mV, i.e., only the EPSP amplitudes ≥27 mV elicited APs. Thus, the EPSP amplitudes sufficient to cause output spikes in the absence of strong inhibition were now subthreshold. The inhibitory strength was then systematically varied according to the static and to the dynamic synaptic mechanism models (Figures 7C–F). For both conditions, increasing the initial inhibitory conductance consistently raised the threshold EPSP (Figure 7C). In unison with results in Figure 6, the inclusion of the dynamic synaptic mechanism reduced the impact of the initial decay time constant. Therefore, the EPSP threshold only depends on the initial decay time constant in the static model (Figure 7D). A 2D plot depicting the threshold EPSP against varying combinations of inhibitory conductance and decay time constant, further confirms this result (Figure 7E). Addition of inhibitory synaptic plasticity slightly reduced the threshold EPSP values (Figure 7F; note predominance of colder colors). Still, the strength of inhibition had a major influence on the threshold EPSP.

Together, these results corroborate our previous studies demonstrating that inhibition may act by increasing the threshold EPSP in SBCs, thereby allowing only the strongest input events to cause output spikes (Kuenzel et al., 2011; Nerlich et al., 2014a). According to the present findings, the input rate determines the magnitude of inhibitory summation and the resulting increase in threshold. Hence, the inhibitory input sets the threshold EPSP in an input-dependent manner and, therefore, dynamically determines the input-output function of SBCs.

### Effect of Inhibition on the SBC Model Output: Phase Locking

In order to evaluate the effect of inhibition on the temporal precision of the SBC-model output, simulated AN fiber activity upon pure tone stimulation at CF was used as the model input. The simple AN-model produced robust phase-locked spiking (144 ± 1 sp/s; VS = 0.87; phi = 0.33). The synaptic mechanisms of the SBC model were driven by the phase-locked spiketimes (Figure 8A). With minimal inhibitory conductance (1 nS), the output of the SBC model showed lower phase-locking precision (VS = 0.72) and a phase delay with respect to the input (phi = 0.03). Furthermore, phase-locking precision was even worse with strong inhibitory conductance (23.8 nS, VS = 0.62) and the mean phase delay was greater (phi = 0.17). Systematic increment of the inhibitory conductance revealed gradually more impaired phase-locking precision and increasing phase-delay (Figures 8B,C). The effects were similar for the static and the dynamic synapse mechanism model (Figure 8B). However, the phase delay was less prone to change in the static synapse model (Figure 8C).

The cycle histograms and vector-strength values presented here were calculated by using the peak of the AP as spiketime of the SBC output. This was considered the most relevant metric for the SBC function. Further analysis of the temporal precision between the peak of the EPSP component (successful EPSPs only) and the following AP revealed the cause of the phase-locking deterioration. The EPSP timing had vector strength of 0.81 ± 0.01, i.e., on average 0.15 ± 0.02 better than the phase locking of the AP. Moreover, the precision of the EPSP peak was not affected by the inhibitory strength and the mean phase delay was only slightly increased (phi = 0.72 for minimal inhibition, phi = 0.79 for maximal inhibition). AP initiation close to threshold, presumably occurring under the influence of strong inhibition, was thus the major cause of poor temporal precision in the SBC model. Thus, these data suggest that the interaction of excitation and inhibition *per se* reduces the temporal precision of the SBC model mainly by causing AP initiation close to threshold, which introduces a non-linear temporal delay to the events. As shown in Figure 7, increasing inhibitory strength raises the threshold EPSP, thereby causing otherwise strong events to become just suprathreshold.

The present modeling results corroborate earlier findings from *in vivo* recordings indicating that inhibition alone is not sufficient to improve the temporal precision of SBC. Kuenzel et al. (2011) however showed that large EPSP selected by high thresholds were endowed with higher temporal precision. Based on this, it was hypothesized that inhibition helps to improve phase-locking precision by preferentially selecting such large and well-timed events. Although the mechanism underlying the relation between EPSP size and temporal precision remains elusive, this hypothesis was tested in the improved SBC model. To this end the EPSG were weighted by their phase distance to the mean phase (phi) of all AN spikes. After weighting, poorly timed events that contribute most to reduced phase-locking accuracy were up to 50% smaller than events generated at preferred phase of the AN fiber (Figure 8D). We used a condition of reduced input phase-locking precision to emphasize the improvement by the SBC (AN at 1/3 octave below CF VS = 0.69, phi = 0.79). When paired with a weak inhibition of 1 nS a small improvement of phase-locking precision was observed (SBC VS = 0.71, phi = 0.37) for both the static and the dynamic synapse mechanism model. Further increment of the inhibitory conductance up to 9 nS slightly increased the VS for both synaptic models (Figure 8E). Thereafter, stronger IPSG decreased the VS of the static model, whereas the phase locking in the dynamic synapse model showed further gradual improvement. In the latter model, the SBC output is clearly better phase-locked than the input, showing a VS increase >0.1 at inhibitory conductances >20 nS (Figure 8E). This behavior is qualitatively and quantitatively comparable to *in vivo* SBC recordings from gerbils where the relative output vs. input vector strength was raised in conditions of high failure rate (Kuenzel et al., 2011).

The second parameter used to quantify phase-locking accuracy, the phase shift of the output spike, showed distinct profiles in both synaptic models in dependance on inhibitory strength (Figure 8F). In the static synapse model, the mean phase of the output was bell shaped, revealing maximum phase change at the IPSG of 12 nS. Such relationship between the phase delay and inhibitory strength could be detrimental for phase dependent coding. On the other hand, the dynamic synapse model showed a monotonic behavior, i.e., a longer phase delay for inhibitory conductance that also induced prominent vector strength changes (cf. Figure 8E).

In summary, our data suggested that tonic inhibition does not increase temporal precision *per se*. However, when acting together with a putative phase-dependent modulation of excitation, tonic inhibition assumes a critical role in temporal coding of SBC.

## Discussion

*In vivo* recordings in the AVCN consistently revealed non-monotonic rate-level tuning in a large proportion of SBCs (Winter and Palmer, 1990; Kopp-Scheinpflug et al., 2002; Dehmel et al., 2010; Kuenzel et al., 2011). In this study, we determined the inhibitory parameters necessary to reproduce such input-output relationship in a SBC model. Implementation of inhibitory properties derived from slice recordings in the model provided a close match to *in vivo* data from anesthetized gerbils. The present results demonstrate that slowly decaying, strong inhibition that summates to tonic levels in an activity dependent manner optimally reproduces the recorded *in vivo* data in the model. Notably, this only applies to a defined range of excitatory and inhibitory conductances, consistent with values measured in acute slices. We therefore suggest that similar conductance levels are probably active *in vivo*. Surprisingly, the interaction of inhibition and excitation primarily reduced phase-locking precision in the model. Thus, inhibition alone may not suffice for an improvement of temporal precision. However, inhibition increased phase-locking precision when the EPSP size was dependent on the phase of AN fiber discharges. Hence, the high-pass filter property of synaptic inhibition acts by selecting the largest and best phase-locked input events. The activity dependent dynamics of the inhibitory inputs seems necessary to cause a stable and predictable output, presumably by emphasizing the tonic nature of the inhibition acting on the SBC.

### Factors Contributing to Non-Linearity of the Input-Output Function

By exploring the effects of a wide range of model parameters on the SBC output functions and evaluating similarity between the model and the recorded data, we effectively used a simple fitting approach to assess the inhibitory synaptic parameters active *in vivo*. A comparable approach was recently used by Fontaine et al. (2013) to predict the exact temporal structure of the spiking response of an integrate-and-fire model. Correlating the respective model to recordings performed in the auditory system corroborated that spike-threshold adaptation is a critical component of the SBC output function. Refractory period at short intervals, and interval-independent increase of the AP threshold due to inhibition were previously suggested as the main factors determining the input-output relation (Kuenzel et al., 2011). Our present data further elaborate this notion by showing that synaptic inhibition contributes to threshold adjustment in a stimulus-dependent manner. In the present model, the biophysics of the SBC and the interaction of excitatory and inhibitory synaptic conductances were taken into account as the factors determining the shape of the RLF. Although such model has limitations due to necessary simplification, it allowed us to assess a large number of possible interactions and to estimate the conductance parameters generating a RLF as shown in Figure 1C, i.e., excitatory conductance of 55–62 nS; inhibitory conductance of 7–17 nS, depending on the excitatory synapse model.

Studies in rodent acute slices investigated short-term depression at the endbulb of Held SBC synapse in great detail during development and in maturity (Bellingham and Walmsley, 1999; Wang and Manis, 2008; Yang and Xu-Friedman, 2008, 2009; Wang et al., 2010, 2011). However, the significance of synaptic depression at the giant calyceal synapses *in vivo* is still unclear, as their release probabilities might differ between *in vitro* and *in vivo* conditions (Borst, 2010). Several lines of evidence raised doubts about a role of short-term depression or suggested chronically depressed synapses *in vivo* (Hermann et al., 2007; Crins et al., 2011; Kuenzel et al., 2011; Klug et al., 2012). The inclusion of short-term depression of the endbulb in our simulations yielded a very poor match to the model data. This corroborates our previous studies, where we found no indications of short-term depression *in vivo*. The potency of inhibition to block APs was investigated in a model driven by a fixed-, stochastically varying-, and dynamically depressing-excitatory conductance (Nerlich et al., 2014a). Here the interaction of inhibition with stochastically varying excitatory conductance provided the best match to the fidelity of the endbulb of Held SBC synapse measured *in vivo*. Still, the effective excitatory conductance range at the endbulb of Held *in vivo* remains unknown. Based on the present data, we propose at least 55 nS, consistent with the endbulb conductances assessed with slice recordings (e.g., Pliss et al., 2009).

### Slow vs. Fast Inhibition

What is the physiological relevance of the activity-dependent dynamics of inhibition, as observed in slice recordings, in the intact brain? Due to slow transmitter clearance enabling glycine spillover and the activity-dependent asynchronous release of glycine and GABA, the IPSC kinetics are dynamically adapted to provide slow inhibition to SBCs during physiologically relevant activity (Nerlich et al., 2014b). Several inhibitory synapses constituting auditory brainstem circuits employ transmitter spillover to nearby and/or extrasynaptic receptors, intersynaptic transmitter pooling and rebinding (Balakrishnan et al., 2009; Tang and Lu, 2012), and asynchronous delayed release (Lu and Trussell, 2000; Tang and Lu, 2012). Recent studies in the AVCN consistently showed slow IPSCs in bushy cells, as opposed to significantly faster inhibition in stellate cells (Xie and Manis, 2013, 2014; Nerlich et al., 2014a). This difference can have a potentially important functional consequence: Compared to the timescale of excitatory events, inhibition is very slow and its efficacy crucially depends on the input rate. Therefore, the tuning of units that provide respective inputs is likely to determine the overall strength of inhibition. To date, the D-stellate cells in the AVCN and the tuberculoventral cells in the DCN (Wickesberg and Oertel, 1990; Campagnola and Manis, 2014) have been identified as the major sources of inhibition to SBC. Both neuron types receive primary inputs from the AN. Inhibitory units in the deep DCN have been described as “type II” response units (cf. Young and Voigt, 1982; Spirou et al., 1999), which are characterized by low or no spontaneous activity, narrow frequency tuning and moderate non-monotonic RLF tuning. With the last characteristic not being implemented into our model, some overestimation of the rate of inhibitory events at high sound pressure levels may be possible. However, due to the long decay time constant of the IPSG and consequently strong summation already at moderate rates, we deem the impact of this to be minor for the outcome of the model.

### High-Pass Filter Properties of Inhibition

The non-monotonic rate-level tuning is determined by the integration of sensory inputs in the SBC. At high sound pressure levels, the reduction of the output rate with respect to the ANF input (Kopp-Scheinpflug et al., 2002; Kuenzel et al., 2011) strongly suggests the effect of stimulus-dependent inhibition. Notably, *in vivo* recordings provided evidence for high phase-locking accuracy of the remaining spikes (Kuenzel et al., 2011). Implementation of inhibitory parameters that generate a non-monotonic RLF in our model decreased the temporal precision of the remaining output spikes (Figures 8A–C). This is consistent with an inhibitory strength that renders many synaptic events subthreshold. Generally, AP initiation close to threshold is considered to introduce a temporal jitter due to the stochastic nature of sodium channels (Steinmetz et al., 2000; Gittelman and Tempel, 2006). This would cause poor phase locking at higher frequencies. Still, *in vivo* recordings showed that high EPSP precision efficiently compensates for AP jitter occurring in conditions with strong inhibition. Our model corroborated this when the phase-dependent amplitude of synaptic events was implemented. The rationale for this approach was based on the *in vivo* recordings indicating the phase dependency of the endbulb EPSG, but the underlying mechanism remained unknown (Kuenzel et al., 2011). An appealing hypothesis is the convergence of inputs, with exact coincidence of more than one input producing a stronger and steeper EPSP that rapidly and predictably crosses the threshold and triggers a spike. This is also plausible for few synaptic inputs, as investigated theoretically by Xu-Friedman and Regehr (2005). However, in low frequency SBC showing high phase-locking precision, analysis of EPSP amplitudes *in vivo* did not reveal a multi-modal amplitude distribution that would be expected to result from the convergence of few large inputs (Kuenzel et al., 2011). We hypothesize that coincidence of a single large endbulb input with smaller AN synapses on the dendrites of SBC (Gómez-Nieto and Rubio, 2009) might “boost” the excitation to achieve the effect we propose.

In summary, this study reveals the functional importance of dynamic inhibitory filtering in sensory neurons and determines the inhibitory parameters putatively active *in vivo*. Applying the same approach to simulate complete frequency-response maps while referring to the differential frequency tuning of presumptive inhibitory inputs (on-CF vs. sideband inhibition), will provide a comprehensive picture of inhibitory contribution to the SBC output function.

## Conflict of Interest Statement

The authors declare that the research was conducted in the absence of any commercial or financial relationships that could be construed as a potential conflict of interest.
